# Spirituality During the COVID-19 Pandemic: An Online Creative Arts Intervention With Photocollages for Older Adults in Italy and Israel

**DOI:** 10.3389/fpsyg.2022.897158

**Published:** 2022-06-16

**Authors:** Shoshi Keisari, Silvia Piol, Hod Orkibi, Talia Elkarif, Giada Mola, Ines Testoni

**Affiliations:** ^1^School of Creative Arts Therapies, University of Haifa, Haifa, Israel; ^2^The Centre for Research and Study of Aging, University of Haifa, Haifa, Israel; ^3^The Emily Sagol Creative Arts Therapies Research Center, University of Haifa, Haifa, Israel; ^4^Department of Philosophy, Sociology, Education and Applied Psychology (FISPPA), University of Padova, Padua, Italy

**Keywords:** spirituality, older adults, creative arts therapies, online interventions, COVID-19 lockdowns

## Abstract

The present study aimed to examine how expressions of spirituality were stimulated and reflected in an online creative arts intervention for older adults during COVID-19 lockdowns. The online process focused on the creation of digital photocollages together with narrative elements of dignity therapy. Twenty-four Israeli and Italian community-dwelling older adults aged 78–92 participated in a three-session online intervention involving the production of three photocollages. The visual and verbal data (participants’ chosen photos and photocollages, and transcripts of the sessions) were qualitatively analyzed within an abductive framework. Four themes were generated, representing the four domains of spirituality that were stimulated by and expressed in the process: (1) Connectedness with the self, (2) connectedness with others, (3) connectedness with the environment, and (4) connectedness with the transcendent. The findings show how photographs can serve as projective visual stimuli which elicit personal content through spontaneous thinking, and they reveal the multifaceted nature of spirituality, wherein each domain nourishes the others. Overall, the findings illustrate how creative arts intervention guided by the tenets of dignity therapy can contribute to the spiritual care of older adults during periods of social isolation, or to the spiritual support provided in palliative care.

## Introduction

Social restrictions introduced in response to the COVID-19 pandemic have caused many people psychological distress (Zhu et al., 2021; Keisari et al., 2022a). Social distancing mandates particularly affected older adults who live alone or rely on psycho-social support outside the home (Morrow-Howell et al., 2020; Steinman et al., 2020). The pandemic also led to a parallel outbreak of ageism, with an increasing portrayal of older adults as helpless, frail, and unable to contribute to society (Cesari and Proietti, 2020; Ayalon et al., 2021). Exposure to ageism is associated with a decline in self-esteem and dignity (Lev et al., 2018) and with poorer mental health (Lyons et al., 2018). The combination of isolation and ageism thus created a substantive need to support older adults and enhance their coping resources in this time of crisis (Wand et al., 2020; Wu, 2020). Studies point to the value of spirituality in promoting wellbeing and as a coping resource (Reutter and Bigatti, 2014; Lifshitz et al., 2019). Recent studies on mental health, spirituality and COVID-19 show that spirituality contributes to wellbeing and resilience in times of adversity (Prazeres et al., 2020; Del Castillo, 2021; Zhang et al., 2021).

This study reports an online creative arts intervention for older adults implemented during COVID-19 lockdowns in two countries, Italy and Israel, both of which instituted strict social distancing measures and curfews to mitigate the spread of the virus. The life expectancy in both countries is similar (in 2020 the life expectancy in Italy was 84.8 years for women and 79.7 for men and in Israel 84.8 years for women and 80.7 for men). However, the older population in Italy (age > 65) makes up a larger percentage (about 23.5% of the whole population) than in Israel (roughly 11.95%) (Central Bureau of Statistics, 2021; ISTAT, 2022). Another major difference between the two populations has to do with religiosity. While in Italy the majority of the population describe themselves as Christian (for example 79.2% of the population are Catholic; European Union, 2021), the population in Israel is mostly Jewish (for example 74% are Jewish and 18% are Moslem; Central Bureau of Statistics, 2021).

Creative arts therapies (CAT) is an umbrella term for healthcare professions employing the creative and expressive process of art-making to foster psycho-social wellbeing (Shafir et al., 2020). A growing body of research points to the value of creative art interventions for older adults, and their ability to improve various facets of mental health such as meaning in life, self-acceptance, wellbeing, positive relationships with others, and the reduction of symptoms of depression (Beard, 2012; Ciasca et al., 2018; Dunphy et al., 2019; Keisari et al., 2020). However, there is as yet scant literature on the ability of creative arts therapies to provide spiritual care for older adults. The current study aimed to help bridge this gap.

Our intervention combined a short-term individual creative arts therapy project, in which participants created their own photocollages, with narrative elements of dignity therapy, a psychotherapeutic mode aimed at helping individuals approaching the end of life maintain their dignity and sense of purpose (Chochinov et al., 2004, 2005). In this research, we sought to understand how engagement in the creative process, guided by the tenets of dignity therapy, helped participants connect to their spirituality as a coping resource. In so doing, we drew on theories that explore the dimensional structure of spirituality (Fisher, 2010; de Jager Meezenbroek et al., 2012) and its relationship with wellbeing in later life (Thauvoye et al., 2018; Lifshitz et al., 2019). This study examined how the spiritual care provided was manifested in four domains or orientations: connectedness with the self, connectedness with others, connectedness with the environment or nature, and connectedness with the transcendent.

### Spirituality in Late Life

Before we proceed, let us clarify what we mean by spirituality. Social scientists draw a distinction between spirituality and religion, where the latter tends to refer to the external, institutionalized, formal, and doctrinal ways by which people engage with some concept of the sacred, transcendent, or divine (Dalby, 2007). Spirituality, by contrast, refers to personal, subjective, existential experiences (Gijsberts et al., 2011; de Jager Meezenbroek et al., 2012).

Theories of spirituality emphasize the human need to search for meaning and purpose in life (Muldoon and King, 1995; Daaleman et al., 2004; Emmons, 2006), and the universal desire to feel “at peace” (Steinhauser et al., 2006). These theories highlight how spirituality, especially in secularized Western societies, allows people to feel the divine within themselves, rather than as an external higher entity (Heelas, 2000), or link spirituality to a sense of connection with the essence of life (de Jager Meezenbroek et al., 2012).

There is a general consensus that spirituality is multidimensional in nature (Puchalski, 2012; Steinhauser et al., 2017). de Jager Meezenbroek et al. (2012), for example, described four dimensions of spirituality: Connectedness with transcendence as a higher being, connectedness with the self, connectedness with others, and connectedness with nature. Similarly, Fisher’s (2010) model of spiritual health and wellbeing recognizes four domains: (a) The personal domain, which deals with self-awareness as a source of meaning, purpose and values in life; (b) the communal domain, which concerns the quality and depth of interpersonal relationships; (c) the environmental domain, which relates to the notion of unity with the environment; and (d) the transcendental domain, which relates to one’s relationship with a higher entity. Those models both center around different orientations of spirituality. By contrast, Gijsberts et al. (2011), following a systematic review of instruments measuring spirituality in end-of-life populations, distinguish between three measurable dimensions of spirituality: spiritual wellbeing (e.g., peace); the spiritual cognitive behavioral context (e.g., spiritual beliefs, spiritual activities, and spiritual relationships); and spiritual coping.

Spirituality tends to develop during later adulthood, even in secular modern societies (Wink and Dillon, 2002, 2008; Moberg, 2008). Specifically, people tend toward more spiritual perspective from mid-life onward, where spirituality takes on an increasingly central role as people cope with aging and the approaching end of life (Gijsberts et al., 2011). Several explanations have been put forward for the development of spirituality in later adulthood, including awareness of one’s mortality that comes at midlife, and changes of priorities in life, since before midlife, external constraints associated with launching a career and establishing a family tend to be dominant. Other studies suggest that the adversities related to old age, such as the loss of meaningful others, exposure to ageism, and restricted mobility can direct many older adults toward spirituality as a resource (Wink and Dillon, 2002). Tornstam’s theory of gerotranscendence holds that as people age, they undergo a shift in perspective, from a materialistic and rational view of the world to one that is cosmic and transcendental (Tornstam, 1997). Gerotranscendence typically includes a redefinition of the self and relationships to others and a new understanding of fundamental existential questions. It is characterized by an increased feeling of affinity with past generations and a decreased interest in superfluous social interactions. Individuals become less self-concerned and interested in material things, and more selective in their choices of social and other activities (Hyse and Tornstam, 2009).

Studies have found associations between spirituality and various aspects of mental health and wellbeing in old age (McCauley et al., 2008; Reutter and Bigatti, 2014; Malone and Dadswell, 2018). Spirituality contributes for example to promoting and maintaining resilience in later life (Manning, 2013). It is associated with increased life satisfaction over time, and can influence older adults’ interpretations of events, making events seem generally meaningful, and determining whether events are seen as opportunities, rather than threats and demands (Cowlishaw et al., 2013). Spirituality can also act as a buffer against death anxiety (Budhiraja and Midha, 2017). Spirituality is also associated with lower COVID-19-related anxiety (Prazeres et al., 2020). In addition, spiritual fortitude, which refers to the ability to draw on spiritual resources in the face of stressors, is related to various facets of mental health, such as meaning in life, spiritual wellbeing, and perceived posttraumatic growth (Zhang et al., 2021).

Some studies investigating how spirituality may improve mental health in older age (e.g., reducing depressive symptoms) have focused on specific domains or dimensions of spirituality (Rykkje et al., 2013; Han and Richardson, 2015; Thauvoye et al., 2018; Lifshitz et al., 2019). The present study adds to this literature by exploring how different domains of spirituality were stimulated by the current intervention, following the orientational models of Fisher (2010) and de Jager Meezenbroek et al. (2012). This intervention is focused on the creation of digital photocollages together with narrative elements of dignity therapy.

### Dignity Therapy

Dignity therapy (Chochinov, 2002, 2012; Chochinov et al., 2005) is a brief individualized form of narrative psychotherapy, targeted at individuals of any age approaching the end of life (e.g., the terminally ill), as well as the older population (Fitchett et al., 2015). Dignity therapy involves the preparation of a physical *generative document*, which is based on the client’s personal narratives. The process of creating the generative document is structured around eight themes: generativity, continuity of self, role preservation, maintenance of pride, hopefulness, aftermath concerns, and care tenor (Chochinov et al., 2005). The full process is intended to increase people’s sense of purpose and preserve their dignity by giving them the opportunity both to reflect upon their lives, and to turn their life narratives into a resource for others (Chochinov et al., 2004). In the current intervention we related to these themes through three topics, one for each session: (1) Turning points in the personal narrative; (2) personal legacy; and (3) future perspectives. We describe our protocol in detail in the Supplementary Material.

Integrating the use of photographs within dignity therapy has been found to facilitate recollection and to help clients structure their narratives. Photographs serve as a projective stimulus, helping elicit past experiences, themes, and visual expressions that support the creation of the narrative (Testoni et al., 2019b, 2020, 2021). More precisely, visual images in photographs can reveal themes and topics which cannot be expressed by words alone, such as emotions, embodied expressions, and muted or sensitive aspects of personal experience (Weiser, 2018; Testoni et al., 2020, 2021). The photographs used in the therapy may be the personal snapshots or family photos belonging to the individual, or may come from some other source (e.g., stock images or art photos).

### Crafting the Life Stories of Older Adults Through Photocollages

Collage is a popular practice in which participants select various materials and images, compose them, and attach them to a surface (Raffaelli and Hartzell, 2016; Russo-Zimet, 2016; Kaimal et al., 2020). Collage-making is an effective way of helping older adults process their memories and life experiences (Stallings, 2010; Keisari et al., 2022b). The creative process stimulates personal content and memories, while still allowing for a reflective distance that promotes verbalization and communication (Raffaelli and Hartzell, 2016). Collage also enables people to engage in art-making with less perceived threat, because it does not demand high technical skills (Stallings, 2010).

This study focused on the creation of digital photocollages, in which participants selected and composed photographs using software tools. Our previous findings show that the projective stimuli of digital photographs supported older adults’ narratives and engaged them in a more embodied emotional experience. Creation of a digital photocollage can thus serve as a visual generative document for participants’ life-stories (Keisari et al., 2022b). This study examined how expressions of spirituality were stimulated and reflected in this online creative arts intervention for older adults. Specifically, we explored which domains of spirituality were stimulated by the creative process, and expressed in the final created products (the photocollages).

## Materials and Methods

### Sample

Twenty-four community-dwelling older adults took part in the study, including 12 in Italy and 12 in Israel. Participants were recruited through community coordinators, social workers at day centers, and family members. Inclusion criteria for the study were: (1) An age of 78 years or over (i.e., late aging); (2) a Mini-Mental State Examination above 24 (reported by social workers and family members), indicating normal cognitive performance (Woodford and George, 2007); (3) absence of any mental disorder or major depression; (4) vision and hearing sufficient to engage in online conversation; and (5) access to a computer screen large enough to engage in the creative process (smartphone screens were not appropriate).

After receiving initial consent to take part in the study, the researchers conducted an introductory phone call with all participants to explain the study aims and content. Participants provided formal verbal recorded informed consent at the start of the first online session (see below).

The study was approved by the Ethics Committee for Experimentation, University of Padua (confirmation number 0581B1B9C39761AE3C03AD3D93EFDEE9) and by the Ethics Committee of the Faculty of Social Welfare and Health Sciences at the University of Haifa, Israel (confirmation number 366/21). To preserve confidentiality, pseudonyms are used for the participants.

### Crafting Life Stories Through Photocollages: Description of the Intervention

The short intervention involved a series of individual online sessions, in which participants made three photocollages. The sessions were structured around themes drawn from dignity therapy. Each session was focused on the exploration of one theme: (1) ***Turning points in the personal narrative***—significant life events and related themes, roles, and coping resources (McAdams, 2001; Chochinov et al., 2002; Keisari and Palgi, 2017); (2) ***personal legacy***—how the participant wished to be remembered, and what values and lessons they would like to pass on to their loved ones (Chochinov, 2002; Testoni et al., 2021); and (3) ***future perspectives***—desires, thoughts and concerns regarding the future and the end of life (Lang and Carstensen, 2002; Testoni et al., 2015, 2017). The full protocol (Keisari et al., 2022b) and TIDieR checklist (Hoffmann et al., 2014) can be found in Supplementary Material.

The sessions were conducted *via* Zoom, with the “share screen” function operational. At the start of each session, the themes and related questions were introduced by the therapist using as prompts a set of artistic photos, selected in advance for their ability to serve as visual metaphors for that session’s theme (Weiser, 2004). The full pool comprised about 80 photographs, either original images by Israeli photographers Michal Fattal and Yehudit Liberman, or licensed photos obtained from the iStock website.^1^ Participants were asked to view the presented photographs, choose those that most reflected their own personal experiences in relation to the themes raised by the therapist, and arrange them together within a blank space on the screen. This was done using PowerPoint software, which is available on most computers and enables flexibility in the creative process of selecting, cropping, positioning, and titling digital photographs. Participants were also permitted to select and incorporate personal photographs, or other photographs taken from the Internet (all such photos were replaced by licensed photographs from the iStock website for this publication). At the end of the process, the digital photocollages were printed and sent to the participants.

The sessions were conducted by the first (SK), second (SP), fourth (TE), and fifth (GM) authors, with each participant working with one therapist. The first author is a supervisor and drama therapist specializing in clinical gerontology. The second and fourth authors were doing internships in clinical psychology and drama therapy, and the fifth author was a master’s degree student in clinical psychology. Two of the therapists (native Italian speakers) conducted the sessions in Italy, and two (native Hebrew speakers) in Israel. The sessions were conducted as a therapeutic process, so that the relationship with the researchers were therapeutic in nature, as this provided a safe, positive and corrective experience for the participants to promote self-development.

To ensure adherence to the protocol, the first (SK) and sixth (IT) authors conducted five training sessions with the team (2.5 h each) prior to the intervention in order to gain familiarity and practice how to deliver the intervention. In addition, 90-min online supervision sessions were held twice a week during the course of the study by the first author. During these sessions, the student therapists were able to discuss their work and receive professional support. Overall, 22 supervision sessions were conducted.

#### The Setting

The original protocol called for three online sessions for each participant, each lasting approximately 90 min. Some meetings were split into two sessions to accommodate the participants. Overall, 78 sessions were conducted between December 2020 and March 2021, ranging in length from 26 to 120 min, with 37 sessions held in Italy and 42 in Israel. Sessions were held about 2–7 days apart. In addition, participants would occasionally contact the therapist outside the formal sessions, *via* WhatsApp or email, to share thoughts, personal photographs, texts, and ideas they wanted to incorporate into their collage. Some participants were supported by family members or day center staff in their use of technology such as setting up the computer and the Zoom meetings.

### Data Collection and Analysis

All sessions were video-recorded and later transcribed for analysis. The qualitative data collected for the study thus included both a verbal component (the transcribed sessions) and a visual component (each participant’s chosen photographs and photocollages).

The analysis employed abductive reasoning, a pragmatist approach to analyzing qualitative data (Tavory and Timmermans, 2014). Following this approach, we began by drawing observations from the data (both verbal and visual), and then sought to make sense of those observations by examining them against theories and conceptualizations found in the literature (Timmermans and Tavory, 2012; Brinkmann, 2014; Earl Rinehart, 2020). This process led us to narrow our focus to the dimensions of spirituality revealed by the data. Specifically, we asked the following question: which dimensions or domains of spirituality were stimulated by the creative process, and expressed in the final created products (the photocollages)?

After selecting our research question, we conducted a polytextual thematic analysis (Gleeson, 2020) of both the verbal and visual data. The dataset was analyzed by the four researchers who had conducted the therapy sessions, using the Atlas.ti 9 cloud software, which allows for a team of researchers to work and analyze the same data together using a shared code set. The visual data were analyzed in relation to the participants’ perspectives. Accordingly, the analysis focused on the participants’ reflections on the visual images, their description (e.g., in terms of colors, shapes, movements, and texture), and the subjective experiences prompted by the photographs and the final photocollage. In addition, the final art products of photocollage were also analyzed by the research team, who took ongoing notes on the themes during the observation.

In the first step, the four researchers reviewed the data of the verbatim transcriptions along with the visual photograph and photocollage data. The verbal data were produced in three languages: Italian, Hebrew, and English (as three of the Israeli participants were native English speakers, their sessions were conducted in English).

In the second step, the researchers conducted an initial coding of the data. The initial coding was conducted in the original language. The two Italian researchers (SP and GM) coded the Italian data, and the two Israeli researchers (SK and TE) coded the Hebrew and English data.

Initial coding by two researchers served to maintain reflexivity since the researcher who conducted the session as a therapist, had an insider’s perspective on the data, whereas the other had an outsider’s perspective. Both perspectives shed light on the effects of one’s position vis-à-vis the phenomenon (Berger, 2015).

To allow the Italian and Israeli researchers to work together, the codes were labeled and defined in English. During this stage, the four researchers met twice a week to review and discuss sample quotations from the transcripts, to ensure that the code definitions were consistent and appropriately applied to the responses. In cases of disagreement, the researchers discussed the coding process until an agreement was reached. These team consultations during the analysis process served to identify possible projections and content missed by the researchers (Berger, 2015).

In the third step, following the abductive reasoning (Tavory and Timmermans, 2014), after the initial set of codes was created, the researchers examined the codes against the theories and conceptualizations in the literature to identify significant broader patterns of meaning, and the codes were grouped into themes and subthemes. For example, the researcher identified the codes of “sense of contributing,” “couplehood as coping resource,” “generativity,” “friendship as coping resource,” “forgiveness,” “universalism,” “care in the family,” and grouped them into three themes: “Connectedness with significant others,” “a sense of contributing to others,” and “caring for humanity.” In the next step, the researchers created links between these three themes to form one core theme labeled “connectedness with others.” The coding and analysis processes ended when theoretical saturation occurred and new data could not contribute to the theme (Fusch and Ness, 2015).

In the fourth step, the candidate themes were checked against the dataset and refined if needed. At this stage, selected quotations were translated to English to enable the third (HO) and sixth (IT) authors to review the thematic map. Note that the translation was verified by the two native speakers (Italian and Israeli) to ensure that no meaning was lost during the translation process. Finally, in the fifth step, the themes and subthemes were organized to create a model.

## Findings

Participants had a mean age of 83.96 (range: 78–92), with a mean of 84 (range: 78–88) for the Italians and 83.92 (range: 80–92) for the Israelis. Of the whole sample, 58.33% were women, 25% were currently married, and all the participants had children. Also, 58.33% of the sample considered themselves religious. Full demographic details of the sample can be found in Table 1.

**TABLE 1:** Participants’ demographics.

Variables	Israeli participants	Italian participants	Total
Mean age (range)	83.92 (80–92)	84 (78–88)	83.96 (78–92)
Gender	6 Females	8 Females	58.33% Female
Place of birth	3 in Israel; 4 in America; 4 in Europe; 1 in Asia	All were born in Italy	37.5% Had immigrated in their lifetime (only Israeli participants)
Marital status	1 Married; 2 divorced; 9 widowed	5 Married; 7 widowed	25% Currently married
Education	4 Had a high school education; 8 had a college education	6 Had a primary school education; 6 had a high school education	25% Had a primary school education; 41.66% had a high school education; 33.33% had a college education
Religiosity	8 Defined themselves as secular, 4 as religious	2 Defined themselves as secular, 10 as religious	58.33% Defined themselves as religious
Religion	11 Were Jewish; 1 identified as an atheist	All were Catholic	45.83% Were Jewish, 50% Catholic, and 4.16% atheist

**FIGURE 1:**
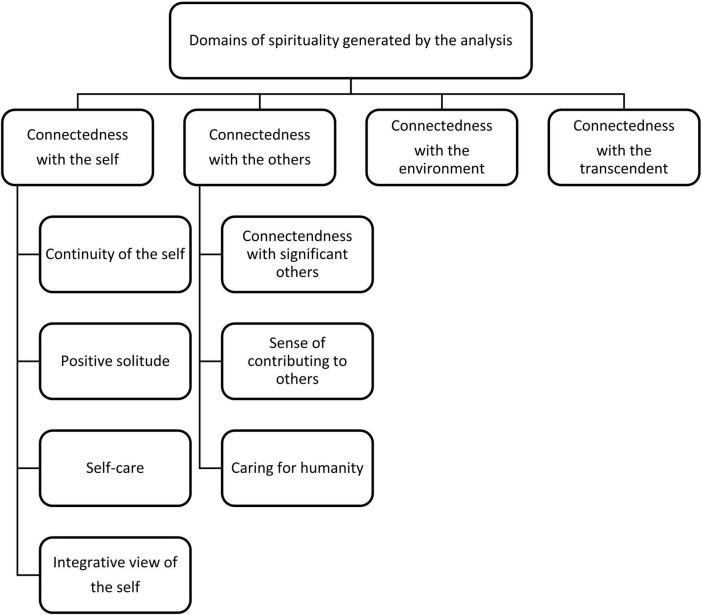
The thematic map.

As described above, our abductive analysis aimed to identify the domains of spirituality that were expressed through the intervention. Four main themes were generated, each representing a spiritual domain or orientation: (1) connectedness with the self; (2) connectedness with others; (3) connectedness with the environment; and (4) connectedness with the transcendent. The first two themes were further divided into several subthemes. The full model of themes and subthemes appears in Figure 1.

In what follows, we introduce and describe each of the main themes and subthemes. Where relevant, we also show how different themes linked together, such that participants’ feelings and perceptions often glided seamlessly from one theme to another.

### Connectedness With the Self

The first theme represents expressions of spirituality that involve connectedness to the self. This theme manifests as an interest in and pursuit of inner knowledge of the self, despite the changes that accompany older age. The theme has four subthemes: continuity of the self, positive solitude, self-care, and an integrative view of the self.

#### Continuity of the Self

The first subtheme refers to participants’ perceived sense of self-continuity in older age, which was stimulated during the creative process. This subtheme was widespread in both the Israeli and Italian samples.

**FIGURE 2:**
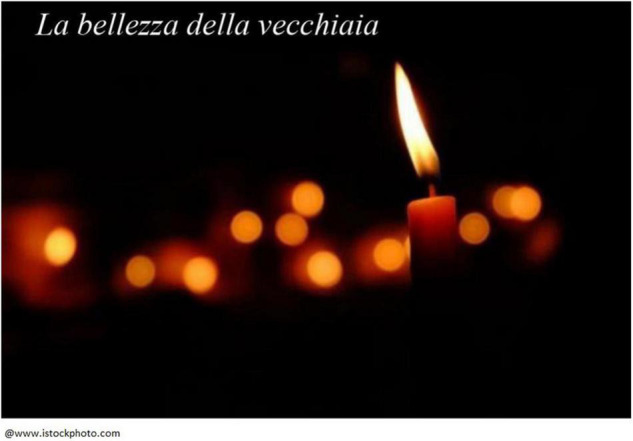
Elena’s chosen photograph and title: “The beauty of old age.”

Elena, an 86-year-old Italian woman, chose the image of a candle (Figure 2) to represent her personal legacy. In describing her choice, she discussed how a sense of self-continuity helps her cope with facing the end of life:

That light over there, it looks a bit like my life. It’s a candle […] that has almost burned down. But it still makes light. And it leaves a trail […] This ray of light, I like it […] What I left behind, the things I’ve done and that I left as something I’ve built. That therefore remains, [it] doesn’t go away […] Good always remains, [the good things you have done] are not lost […] The beauty of life, the beauty of old age. This is the last part of the candle, there is not a candle [anymore]. And the light is vitality. And those glowing red shadows that are behind, I see them as the love I left along the way, and love is the most important thing.

Miriam, an 82-year-old Israeli woman, selected a photograph showing a sheet of music to represent her personal legacy. She explained that the photograph reflects her appreciation of music, a legacy passed down by her parents which remains a key part of her identity. In her photocollage, Miriam positioned this photo next to another one representing a joyful family, and explained: “It looks like the family is dancing.”

Ah, music. plays an important part in my life. Mm. As long as I can remember, I took music lessons. I sang in the choir. I took violin lessons and, you know, and right now, first thing in the morning, I’m going to turn on some jazz, and listen to it peacefully […] I love it, it played a major role in shaping our whole family life. My mother [a Jewish American] always sang in [a local] choir. My father sang in the choir. [Laughing.] My sister played for the choir. We all took piano lessons […] Music is the central, has a central part in my life.

#### Positive Solitude

The second subtheme, positive solitude, refers to participants’ ability to enjoy engaging in activities on their own. Interestingly, the positive solitude theme emerged among the Israeli participants, but none of the Italians. We will return to this difference in the discussion.

**FIGURE 3:**
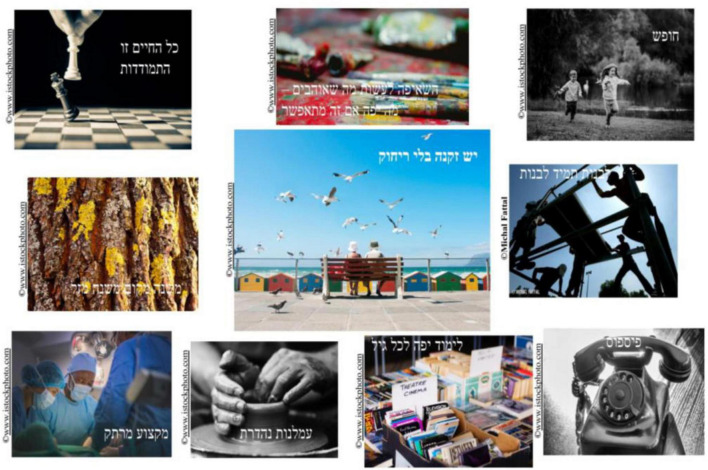
Rivka’s photocollage.

Rivka, an 80-year-old Israeli woman, included photos which represented her love of art, books, and the chess her father used to teach her in her photocollage (Figure 3). While reflecting upon her creation, she discussed how these photos also captured how her positive approach to solitude helped her cope with the COVID-19 lockdowns:

Coping, undoubtedly, there are a few [photos] here. There is “toil.” And “studying” and “chess,” [these] are three of them [pointing to three photos in her photocollage]. […] This means that even in such situations we need to find a way to do things by ourselves. […] I really did not suffer during the whole Corona period […] even though our social life was very very active [before the pandemic] […] I found myself doing things alone and did not get bored for a second this year […] Even though […] I had a foot problem and I had to stay indoors for three months until this problem resolved […] Even in this restricted situation I think I did not cope badly.

#### Self-Care

The third subtheme represents the ability to care for oneself—i.e., to understand what is best for the self and to make life choices that provide one with autonomy. In this study, self-care and the desire for autonomy were identified only among the Israeli participants. Itzhak, an 85-year-old Israeli man, chose the image of a telescope to represent his determination to take care of himself:

I am optimistic. This keeps me going […] Like in this picture with the telescope […] I don’t give up. I try to cope on my own and not be dependent on others. I do laser treatment every day to cope with the pain in my back and shoulder. Every day I do the treatment. I cope. Before I could not lift my arm, now see [shows how he lifts his arm]. Maybe it is the treatment and maybe it is my desire to succeed and to get better results. I am optimistic and I believe that within a month or two I will get to even better results than today.

#### Integrative View of the Self

The fourth and final subtheme under connectedness with the self-concerns an ability to integrate and accept different parts of the self, both positive and negative.

In his first collage, Aron, an 80-year-old Israeli man, composed a row of three photos, which he said symbolized his relationship with his father. One photo represented the disappointment he felt his father perceived in their relationship, while the other two represented love and pride. Aron explained:

One of the things that most disappointed my dad, […] he was also a chess player. He tried to teach me chess. And I was not attracted to it, as he had hoped for, and I know it terribly disappointed him […] so this picture symbolizes disappointment… and here you add the picture with the books, he really liked books and my whole world was books. I read a lot. And he was always proud that I read like this. […] And here is this picture of the porcelain [representing the factory where he worked]… and my father was very proud of his work, and I visited the factory, I was very proud of him and their achievements… So here we have in one line three pictures that actually relate to memories from my dad.

In integrating these contradictory aspects of his relationship with his father—disappointment, love, and pride—in his collage, Aron was able to accept both the positive and the negative as building blocks of his own identity.

Indeed, the use of collage is particularly suited to formulating and expressing an integrative view of the self, since collage itself is a medium in which distinct elements are crafted into a new whole. During the creative process, participants were invited to think about how different visual images might represent different feelings and life experiences. The images participants incorporated into their photocollages often captured contrasts and contradictions—sweet childhood memories along with representations of traumatic events, portraits of newborns next to photos of older adults, images of antiques and old-fashioned handicrafts juxtaposed with images representing modern high technology.

Caterina, an 87-year-old Italian woman, began to reflect on a photo that she saw as representing friendship. This prompted her to speak about her self-acceptance:

I have a lot of friends, people who love me, old friends of mine. They really give me joy and remind me of the fact that, sure, I could have been better in my life, behaved in a better way, but you can see that a lot of things [I did] made other people happy. Then sure, I’ve made a lot of mistakes. And I made mistakes on a lot of things, I can see that, but I’ve always tried to fix them and to feel at peace with myself. […] It’s true, when you get to a certain age, you think about the life you’ve lived. And you say I did this and that wrong. But I did a lot of other things right. I justify it to myself, you know, if I did something wrong.

We can also see here how the spontaneous, creative nature of the collage-making process, and the tangible nature of the visual images that we presented to participants, allowed different domains of spirituality to link up and overlap with each other. In Caterina’s reflections, an image which represented connectedness with others stimulated a more integrative view of herself, in which she was able to accept different pieces of her life.

### Connectedness With Others

The second theme, already mentioned, represents expressions of spirituality that involve connectedness with other people. Three subthemes were identified within this theme: connectedness with significant others, a sense of contributing to others, and caring for humanity.

#### Connectedness With Significant Others

**FIGURE 4:**
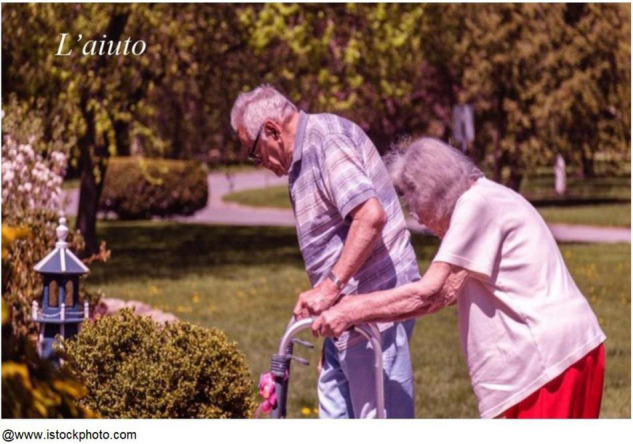
Margherita’s chosen photograph and title: “The support.”

A major subtheme concerned participants’ experiences of love and caring within their most proximal relationships. The most important of these relationships was often with a spouse. Many of our participants reported that couplehood played a major role throughout their lives. Asked to describe how she coped with aging and during the pandemic, Margherita, an 83-year-old Italian woman, chose a photo of two elderly people walking side by side (Figure 4):

A love for life. I’ve been with my husband for my whole life. You know, at the age of seventeen I was already engaged. I got married at 24. It’s been 59 years that we’ve spent together. We are married. Isn’t it beautiful? You know why it’s beautiful? Because there is love. […] Yes, I see a value in this photograph because there is a person who is alive, and that’s one thing, and second thing, you are hugging someone who’s alive too. Then, you walk badly, you have a cane but you have a smile that keeps you going […] being with my husband. Being together, the two of us. […] In this difficult time [the pandemic], being together, husband and wife, pretty old […] It helps me get through this period. […] When you are two, it’s easier to solve things.

**FIGURE 5:**
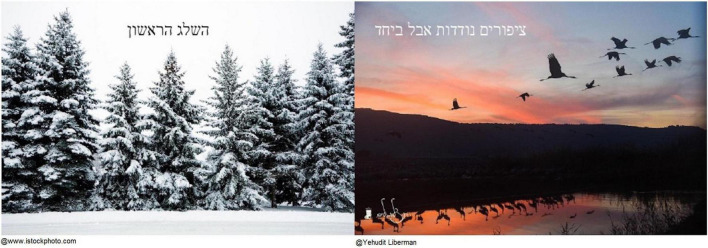
Lea’s photographs and titles: “The first snow” and “Birds migrate but together.”

Another important source of connectedness with others was friendship. Lea, an 83-year-old Israeli woman, described how friendship supported her in her childhood as a Holocaust survivor, and how friendship was important during COVID-19. From a photo of a snowy landscape that recalled her childhood memories as a Holocaust survivor in a boarding school, she shifted to a photo of migrating birds, which she saw as representing a sense of togetherness in life (Figure 5):

I will take this picture, of the snow […] It was in the Italian Alps. I was in a boarding school for Holocaust children like me […] and I remember it snowed […] And we played in the snow, and we were really happy. Snow symbolizes purity, something clean and pure and beautiful. We really had good time in this boarding school […] So, at first I did not open up, I was very closed. But I met a friend there, a little older than me, and we were very good friends. And I really loved her, the way I love myself. What’s happening now [the pandemic], all these fears, the anxieties, about Corona. So, a lot of people close themselves off, I closed myself off a little too, a little too much. [Short laugh.] And I remembered, in life I had many difficulties, during the Holocaust. And it helped me [being with others]. And it’s the same […] [points to the picture of the birds]. And here they [the birds] migrate, migratory birds. Together. And I guess if one is sick, they might help it.

Here again we can also see how the spontaneous flexible nature of the creative process allowed participants to glide smoothly from one domain of spirituality to another, revealing as well how the domains are inextricably connected. The photograph with which Lea began—the snowy landscape—reminded her of her connection to nature, her awe at the beauty of the snow. This intuitively led her to the memory of a good supportive friendship, which she represented by the image of the flock of birds.

**FIGURE 6:**
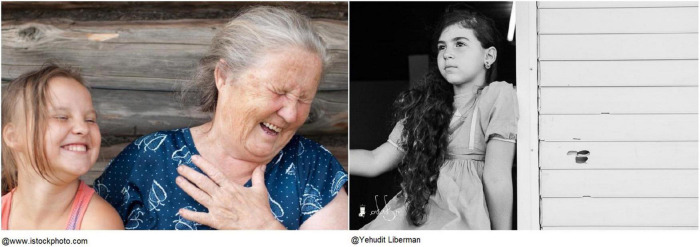
Caterina’s chosen photographs.

Participants’ musings and reflections often suggested the importance of their role as grandparents and their connectedness with their grandchildren. Caterina, an 87-year-old Italian woman, related to two different photographs to describe her relationship with her grandchildren, who infused her with hope for the future (Figure 6). The first depicted a grandmother laughing with her grandchild, and the second a young girl whose gaze reminded Caterina of her grandchildren:

This grandmother [in the photograph] seems almost complicit with this child, she’s got a sweet gaze, and these joys, you have to cultivate them. It’s the wish to have children and them also to have grandchildren to love. Because I notice that when I look at my grandchildren, I don’t care if they are good, I look them in the eyes and I see everything positive. That is, they help me think of a better future, I mean, a future for them […]. This child’s eyes [in the photograph] give me joy, hope for a future for everyone. For everyone, for the world, for us. They give me hope, I don’t know. It’s a feeling I have.

This subtheme also captures participants’ experience of accepting other people, and their ability to forgive others despite previous difficulties in close relationships. Hana, an 87-year-old Israeli woman, talked about her experience of forgiving her mother. When describing this experience, she related to a personal photograph of her mother and titled it “Bella Bellissima”:

Here [looking at her first photocollage], I can see my anger… But from the perspective of time one can no longer be angry. It’s a replica of it [my anger], or a memory of anger. Whoever keeps on being angry at their parents and blames them is wrong. [One] should come to a resolution. So … there’s a picture of my mom above me here. And I call her “Bella Bellissima.”

#### Sense of Contributing to Others

**FIGURE 7:**
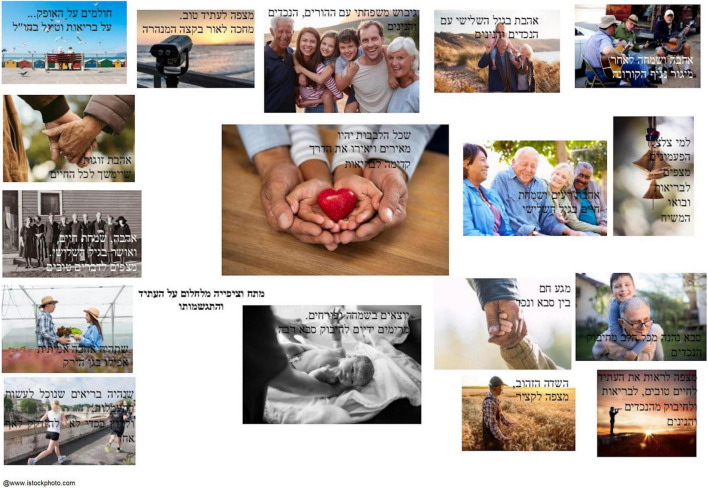
A photocollage by Itzhak: “Tenseness and anticipation while dreaming about the future and its fulfillment.”

Another subtheme found among many participants captures their experience of helping other people, and how this gave them a sense of contribution and meaning in life. Itzhak, an 85-year-old Israeli man, selected a photograph showing two sets of hands, an adult’s and a child’s, cupping a luminous heart as the central image for his photocollage (Figure 7). He said he liked to sew for those in need and that helping others is something that gives him a sense of joy and meaning:

Itzhak: In the center [of the photocollage]. This is it, this is it. Love, well […] the heart. The heart that illuminates our future. Lots of hearts, one heart, one big light.

Therapist: But from the things you have said […] that there will be true love everywhere […] Is there anything that helps these things happen?

Itzhak: I have a sewing machine. That I help people, help those who need. I sew for them […] I sew pillows, masks. Whatever comes […] Whoever comes to me, “Something tore,” and I say “Bring it!” […] I love it, I love to be told “thank you very much.” It is enough. It is joy in the heart. I never take [money]. I do not ask for a penny. I always help. From the heart. And I want more and more and more […] If I’m healthy I’ll do anything.

Giovanni, an 88-year-old Italian man, chose a personal photograph depicting himself as a musician along with other musicians and friends doing volunteer work in a nursing home. He positioned it next to another photograph of musicians and called it “Donating a smile”:

As my hobby, we often went to nursing homes to make them smile… Ever since I went back to music when I retired. It was in the nineties. We were a group of five people. And we went there to have them smile…If it wasn’t for Covid, this would have been my future.

#### Caring for Humanity

The final subtheme under “connectedness with others” represents a sense of connectedness and respect for humanity more generally.

**FIGURE 8:**
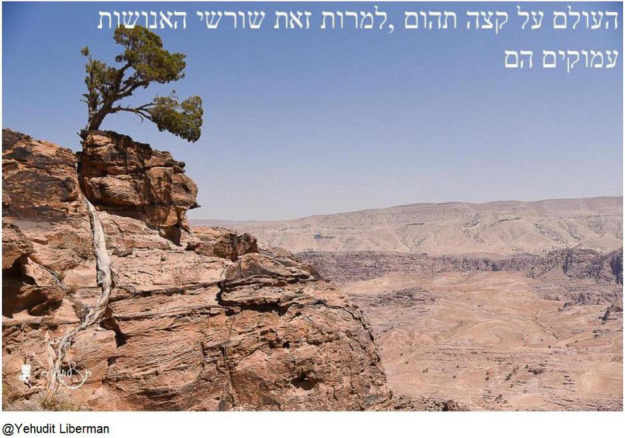
Rivka’s chosen photograph and title: “The world is on the edge of an abyss, yet the roots of humanity are deep.”

Rivka, an 80-year-old Israeli woman, related to COVID-19 when she chose a photograph of a tree growing at the edge of an abyss to represent her hope for humanity (Figure 8):

This is also a beautiful picture, the roots. The picture with the tree and the roots in the rock. A beautiful and interesting picture. It’s like I think the world is on loan, yes? That’s what I say. The world is on the edge of an abyss, but our roots, humanity, perhaps the roots of the founders of the land are deep and strong […] The roots that have grown throughout history are so deep that even on the edge of an abyss […] [She titles the photo] “The world is on the edge of an abyss, yet the roots of humanity are deep.”

Mary, a 92-year-old Israeli woman, chose a photograph of an airplane, which she titled “Openness to culture,” to represent her concern for humanity, her acceptance of other cultures, as part of her sense of universalism as her legacy.

Yes. This one for me is important [the airplane] because it symbolizes travel. And, for me I think it’s important that people, um, have an open mind about other people and other cultures. Because very often people think that yeah, my way is the only way forward. I think it’s important for people to know how many different ways people live […] I think people would get along better if they understood that other people have other ways of dealing with their life. Otherwise, I think a lot of misunderstandings and maybe arguments on being distanced from other people comes from the fact that they aren’t open to see how other people live.

Both Mary and Rivka were prompted by the creative process to articulate profound feelings about the future of humanity. In so doing, the process also helped bolster their dignity by enabling them to pass on their wisdom at the end of life.

### Connectedness With the Environment

The third main theme relates to feelings of connectedness with nature and with other living creatures.

**FIGURE 9:**
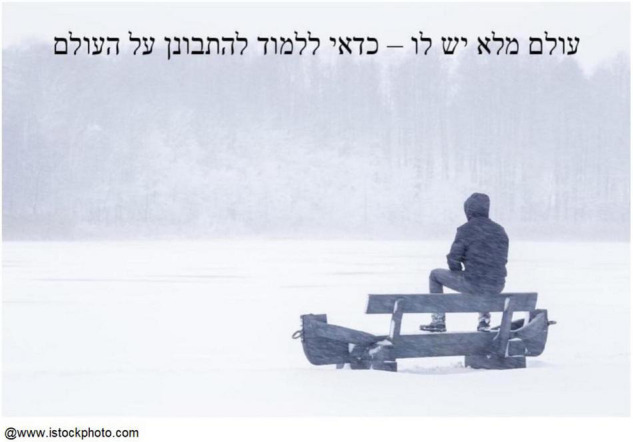
Yossi’s chosen photograph and title: “He has a whole world—it is worth learning to observe the world.”

Yossi, an 80-year-old Israeli man, selected an image of a man, seen from behind, gazing out over a wintry landscape (Figure 9) to represent the value of profound connectedness with the world. The image allowed Yossi to relate COVID-19 as a transient event:

A person looks toward the horizon […] In all this, here he is looking at the sky. He does not look at the sky, he looks at how the future changes every second […] Look, he will be relaxed, sitting, looking at the view. This is a very, very worthwhile thing to learn in life. […] He is looking toward the landscape. He not only looks at a landscape, he looks at how the world grows […] He sees it all. This is a very beautiful thing. a full world he has. He has a full world […] This Corona will be gone, but look at this landscape […].

Many of our participants talked about nature and respect for all living creatures as a coping resource to deal with the COVID-19 pandemic. Miriam, an 82-year-old Israeli woman, put it this way:

I think they should learn the intended lessons of the importance of respecting the environment, all of the live things that surround us, the animals, the trees, the people and the importance of remaining positive in your thinking and your actions […] It’s the only thing that will help cope with current situation [the pandemic]—identifying what caused it and what will fix it. There are lessons to be learned […].

Nature can also symbolize connectedness to other generations and the deceased, as Tova, an 82-year-old Israeli woman, explained. We may note here again how the environmental domain overlaps with the communal. Just as connectedness with nature linked Lea to her memories of friendship, for Tova a connection with nature helps her cope with the loss of her beloved husband:

I’ll tell you a secret, I have a tree by my house, that we planted, me and my husband. And it’s like, I wouldn’t say a tombstone, but for me, it’s my life with him, as if he is still alive within the roots of the tree. And when pigeons come, I speak to them, all the time, as if he is alive. As if he can hear me. And I ask for things. Maybe it is not normal, but it is what it is.

This theme also includes elements of gratitude and appreciation for the simplest things in life. Francesco, an 85-year-old Italian man, when asked to think of a title that would summarize the essence of the three photocollages he created, proposed “The sun gives you the strength to live”:

Because I can’t have a future at my age […] what’s important—I always say—is that if I wake up in the morning, I’m already lucky. […] Personally, I’m greatly in love with life. As a consequence when I see the sun, I’m joyful and I think for a person after 80, 85 years of age everyone’s wish is to wake up in the morning and see a radiant day.

### Connectedness With the Transcendent

The final theme represents spirituality as expressed in connectedness with a transcendent reality or power. The Italian and Israeli participants related differently to this theme. As nearly all of the Italian participants in this study were religious Catholics, many chose images that either had an explicit religious theme or could be interpreted as religious; and they positioned those images more centrally than the Israeli participants. As such, the Italian photocollages often related to faith and religiosity as something that one can cling to during times of adversity. Margherita, an 83-year-old Italian woman, chose a photograph of a church to represent a hope that both she herself and younger generations would keep faith as a coping resource. She called this photo “For a better future”:

You know what helps me [while coping with the COVID-19 pandemic]? […] See, if I go to mass, I come home and I’m happy. I listen to the mass, I resonate, I join the singing […] I’m happy, I don’t know why, but I feel happy. I feel happy when I do that […] I feel like I’ve done something so beautiful. Now I’m used to it but the church for me is everything. […] Because when you have something [to worry about, like the pandemic], you must pray. Because praying gives comfort, even if it does not solve the situation, it comforts you.

Elena, an 87-year-old Italian woman, chose a photograph of a couple looking at the sunset to talk about how she and her husband discovered their spiritual dimension. She explained how faith helped her to cope when her husband was ill in cancer.

Religion can be a sum of rites, of habits. We [my husband and I discovered faith…which brought us to experience a lived spirituality […] and this allowed us to go through a huge trauma when my husband was ill with cancer for a couple of years. […] This allowed us both to be strong and to create an environment of serenity around us and this I owe it, we owe it, to faith. […] The liturgy gives a certain lightness to the heart which is incredible. We derive our strength from it. The strength to live every day with serenity and with a smile. Even during hard times. Since I have been alone, this source of support has been fundamental, as it has allowed me to live my life with a smile.

She later added how her faith helped her to accept the difficulties and restrictions that followed the COVID-19 pandemic. She positioned a photograph of a cross above all the other photos in her photocollage, explaining that it was the most important one and that she wanted it to embrace all the others:

I’m not worried [about COVID-19]. Because, you know, man’s biggest fear is death. This is our destiny, there’s nothing we can do about it. […] With this serenity, I must say, I live. And when the moment comes, I hope I will be able to accept it.

**FIGURE 10:**
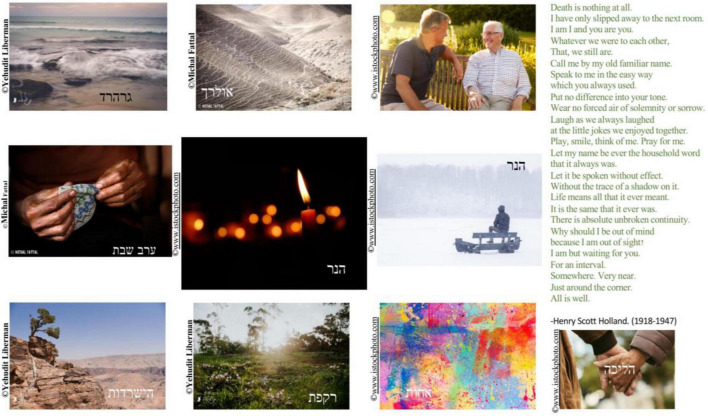
Moshe’s photocollage (untitled).

For the Israeli participants, traditional Jewish rituals and prayers served as a path to the transcendence domain, rather than religious faith itself, as presented by the Italian participants. Moshe, an 81-year-old Israeli man, positioned the photo depicting a candle (Figure 10) to describe how the Jewish ritual of lighting candles on Friday night helped him to cope with the absence of his wife. This is another example, too, of how the domains of spirituality sometimes overlap. For Moshe, a connection with the domain of transcendence created a path to the presence of his deceased wife.

I think the candle [should be in the center]. I light the candles on Shabbat […] and I have a special candle for my wife. With even a special place [for it]. I always light it, in exactly the same color, and it looks the same. So, it gives me a feeling of not being alone […], it’s not that she’s really there or something like that, but I’ve been doing that for a long time, every Friday. Like it’s part of a ceremony […] I’m doing it all by myself [crying]. The candle lighting, the “kiddush” [prayer over wine] [Crying.] […] It’s only my voice inside the living room, when I do the kiddush […] but sometimes I feel her presence […]

Next to this photo, Moshe positioned the photograph of a man gazing out over a solitary landscape and added: “And the part where I sit there alone, put it [in the photocollage] […] next to the candle.” He also asked us to include the poem by Holland (1910) in the collage, to further represent and reflect the continuous bond with his deceased wife.

**FIGURE 11:**
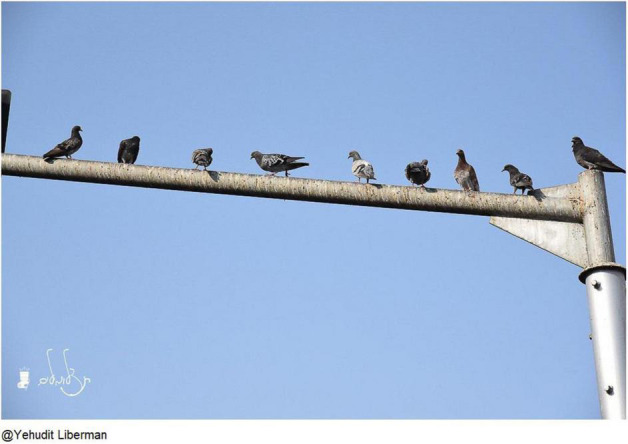
Noah’s chosen photograph (untitled).

Noah, a 91-year-old Israeli man, chose a photograph of perching birds (Figure 11) to represent his appreciation of life and nature. He also linked this image to a Jewish prayer which expresses gratitude for being alive:

Very beautiful, very beautiful. The birds, they don’t ask themselves what tomorrow will be. Someone could come and shoot the bird and it would be gone […]. It’s to appreciate being alive. […] Like in the Jewish morning prayers we say thank you to God for returning our souls to life. To be grateful.

## Discussion

This study developed and examined an online creative arts intervention aimed at helping older adults connect to their spirituality, and thereby support their sense of dignity and their coping resources at a time of social restrictions imposed by the COVID-19 pandemic. The intervention focused on the creation of photocollages integrated with narrative elements of dignity therapy (Chochinov, 2002). We examined how the creative process connected the participants to different domains of spirituality by exploring their life experiences, values, and future perspectives through the creation of three photocollages.

**FIGURE 12:**
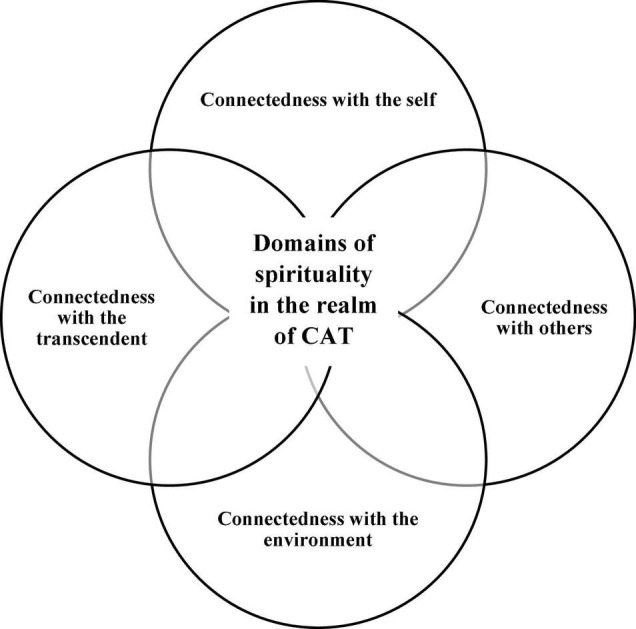
Visual representation of the interconnected nature of the domains of spirituality in CAT.

Four themes were generated, representing four domains of spirituality that were stimulated and expressed by the process (Figure 12): connectedness with the self, connectedness with others, connectedness with the environment, and connectedness with the transcendent. We showed how each domain was stimulated and constructed through the visual images (photographs) used in the intervention, the narratives that were prompted by the photographs and the protocol’s themes, and the creative process of selecting and positioning photographs to create a new art product (a photocollage).

The expressions of spirituality generated from the intervention point to the multidimensional nature of spirituality (Fisher, 2010; de Jager Meezenbroek et al., 2012; Puchalski, 2012). The spontaneous nature of the artistic process also revealed how the different domains of spirituality are interlinked. The projective visual stimuli of the photos elicited personal content as a form of “bottom-up” spontaneous thinking (Yaniv, 2018), through which participants’ reflections in one domain prompted and nourished reflections in another (Figure 12). For instance, as we saw with Lea, the photo of the snowy landscape stimulated her connectedness to the environment through her memories of wonder at the snow as a child Holocaust survivor, and by extension it stimulated her connectedness to others when it aroused the memory of a good friendship at that time of adversity. In other cases, different domains of spirituality actually overlap, especially where the older person has lost a significant other, such as a spouse. For example, Tova and Moshe found that connectedness with the environment (Tova) or with the transcendent (Moshe) helped provide comfort and a sense of remaining connected with the absent other. This observation is consistent with other studies’ findings of correlations between the domains of spirituality (Lifshitz et al., 2019).

The findings suggest that in most respects, the Italian and Israeli participants relied on the different domains of spirituality in similar ways, both when coping with the pandemic and when facing other struggles of life, including the challenges of aging. However, there were several differences between the two groups. In particular, the Italian participants in this sample were all Catholic, and most of them considered themselves to be religious. This was reflected in the Italian photocollages, in which religious images were more often positioned centrally. The Israeli participants, who tended to be more secular, were more likely to refer to their connectedness with and reliance on the self, and to point to positive solitude and self-care as coping resources.

### Current Findings and the Literature

The expressions of spirituality stimulated by the creative process capture the multidimensional nature of spirituality as shown in the literature (Fisher, 2010; de Jager Meezenbroek et al., 2012; Puchalski, 2012). This is one of the first studies to use visual artistic methods to explore these dimensions with the older population. The findings show how photographs, as projective stimuli, allow for intense spontaneous expressions of spirituality in a manner that is flexible and responsive to different needs and perspectives. This is important, because other studies’ findings show that maintaining a connection to different dimensions of spirituality is associated with better mental health and coping in times of adversity, both in general (Whitehead and Bergeman, 2012; Thauvoye et al., 2018; Lifshitz et al., 2019) and specifically during the COVID-19 pandemic (Ribeiro et al., 2020; Lucchetti et al., 2021; Keisari et al., 2022c). The ability of the intervention to connect the participants to the various domains of spiritually as coping resource is crucial, since studies have shown that the COVID social distancing restrictions caused psychological distress for many, including more symptoms of depression (Fountoulakis et al., 2022; Keisari et al., 2022a).

The current findings contribute to the growing body of studies that use photographs with older participants to prompt greater expression and deeper exploration of the participants’ thoughts, feelings and experiences (Kohon and Carder, 2014; Tishelman et al., 2016; Platzer et al., 2021). While most studies in the field use the *photovoice* method, which refers to the participants’ own production of photographs in relation to the research topic (Baker and Wang, 2006; Woda et al., 2018; Chui et al., 2020), or *photo elicitation*, which refers to the use of photographs in qualitative interviews to encourage participants to reflect on particular topics (Platzer et al., 2021), the current study with photocollage uses both ways: the photographs served as projective stimuli to elicit the personal content, and as raw material collected by the participants to make their own artistic product. This stimulated personal content, while also capturing and containing it in a concrete artwork that the participants could then create, observe and reflect upon (Keisari et al., 2022b).

The differences that were found between the Italian and Israeli participants in this sample mirror the familiar and well-established observation that within secular Western societies, people are likely to search for meaning in life within themselves, rather than on the basis of external rules or a connection with a higher entity (Heelas, 2000; de Jager Meezenbroek et al., 2012). These findings also point to the importance of considering intercultural differences in spirituality, and in the dominance of different spirituality domains (Thauvoye et al., 2018; Lifshitz et al., 2019), when designing interventions aimed at offering or strengthening spiritual care.

To the best of our knowledge, this is one of the first studies to develop an online creative arts intervention for older adults, which can be conducted during periods of social distancing (Kordova and Keisari, 2020; Feniger-Schaal et al., 2022). As such, the method goes beyond the current COVID-19 pandemic, as it can also be applied in other circumstances that entail social isolation—for example, among individuals who are homebound or have limited mobility (Milaneschi and Penninx, 2014). This creative online intervention can also be implemented in palliative care, as it is based in part on dignity therapy, known to make a key contribution to the wellbeing of individuals at the end of life (Chochinov, 2012; Fitchett et al., 2015; Testoni et al., 2019a). The current findings are also in line with studies indicating that interventions based on dignity therapy support the existential tasks faced by the majority of older adults, such as settling relationships, and preparing legacies of memory and shared values, as well as preparing for the end of life (Fitchett et al., 2015; Scarton et al., 2018).

Finally, the research methods and findings presented here can enrich narrative therapy protocols for the aging population, such as dignity therapy (Chochinov, 2002) and life review (Westerhof and Slatman, 2019). We found that both engaging with the individual images in the photographs, and the act of meaningfully positioning them in photocollages, helped participants find spiritual meaning in their personal narratives. Thus, narrative therapy protocols could benefit from being expanded to include the visual and creative elements of photocollage.

### Limitations and Future Research

By definition this study consisted of a sample of older adults with access to the Internet and videoconferencing applications. This sample is thus not representative of other groups of older adults experiencing high levels of social isolation during the COVID-19 period, such as nursing home residents, individuals experiencing cognitive decline, or those lacking access to the Internet. The short-term nature of the intervention is also a concern. As mentioned, most of the older adults in the current study were living alone during the social restrictions of COVID, and a longer-term process might have been more beneficial for them.

Future research would benefit from replicating the current intervention with various aging populations, such as older adults with dementia, and in different care settings, such as nursing homes. It is also important to examine the experience of participating in a long-term intervention as a creative process that encourages learning and growth over time. Future studies might also examine the effect of longer-term creative online interventions on various wellbeing measures.

## Conclusion

The objective of this study was to explore how an online creative arts intervention involving the creation of photocollages promoted spiritual care in older adults, and which domains of spirituality were stimulated and expressed. The findings highlight the importance of considering the multifaceted nature of spirituality when using online creative arts therapies to help older adults cope at time of adversity. More broadly, the findings suggest that online creative arts interventions are valuable for older adults during periods of social isolation.

## Data Availability Statement

The datasets presented in this article are not readily available because it contains potentially identifying and personal information. Therefore, the data will be kept confidential under the researchers’ control. Requests to access the datasets should be directed to the corresponding author SK.

## Ethics Statement

The studies involving human participants were reviewed and approved by the Ethics Committee for Experimentation, University of Padua (confirmation number 0581B1B9C39761AE3 C03AD3D93EFDEE9) and by the Ethics Committee of the Faculty of Social Welfare and Health Sciences at the University of Haifa, Israel (confirmation number 366/21). The patients/participants provided their written informed consent to participate in this study.

## Author Contributions

SK and IT conceived, designed the study, and supervised the research team. SK, SP, TE, and GM conducted the intervention, collected, and analyzed the data. SK and SP took the lead in writing the manuscript. HO and IT reviewed the thematic map and the manuscript and helped to improve it. All authors contributed to the article and approved the submitted version.

## Conflict of Interest

The authors declare that the research was conducted in the absence of any commercial or financial relationships that could be construed as a potential conflict of interest.

## Publisher’s Note

All claims expressed in this article are solely those of the authors and do not necessarily represent those of their affiliated organizations, or those of the publisher, the editors and the reviewers. Any product that may be evaluated in this article, or claim that may be made by its manufacturer, is not guaranteed or endorsed by the publisher.

## Footnotes

1
https://www.istockphoto.com/
