# Supplementary material for: Spirituality During the COVID-19 Pandemic: An Online Creative Arts Intervention With Photocollages for Older Adults in Italy and Israel
Source: Front Psychol. 2022 Jun 16;13:897158. doi: 10.3389/fpsyg.2022.897158 (PMC9245519; doi:10.3389/fpsyg.2022.897158)
Supplement: Supplementary file 1 [file Data_Sheet_1.PDF]

TIDieR
Template for Intervention Description and Replication

The TIDieR (Template for Intervention Description and Replication) Checklist*:

Information to include when describing an intervention and the location of the information

Item number	Item	Where located ** Primary paper (page or appendix number)	Other † (details)
1.	**BRIEF NAME** Crafting Life Stories through Photocollages	Page 5	______
	**WHY**		
2.	This online integrative intervention is based on the creative process of digital photocollages together with narrative elements of dignity therapy: ***Dignity Therapy*** is a brief individualized form of narrative psychotherapy, targeted at individuals of any age approaching the end of life (e.g., the terminally ill), as well as the older population (Chochinov, 2002, 2006, 2012; Chochinov et al., 2005; Fitchett et al., 2015). The full process is intended to increase people's sense of purpose and preserve their dignity by giving them the opportunity both to reflect upon their lives, and to turn their life narratives into a resource for others (Chochinov et al., 2004). Integrating the use of photographs within dignity therapy has been found to facilitate recollection and to help clients structure their narratives. Photographs serve as a projective stimulus, helping elicit past experiences, themes, and visual expressions that support the creation of the narrative (Testoni et al., 2020; Testoni et al., 2021; Testoni, Piscitello, et al., 2019). Previous studies show that **photocollage-making** is an effective way of helping older adults process their memories and life experiences (Keisari et al., 2022; Stallings, 2010). The creative process stimulates personal content and memories, while still allowing for a reflective distance that promotes verbalization and communication (Raffaelli & Hartzell, 2016).	The description of the main theories and rationale for the intervention are presented on pages 3-4 and also on page 5.	______ –
	**WHAT**		
3.	The short intervention involved a series of individual online sessions, in which participants made three photocollages. The sessions were structured around themes drawn from dignity therapy. Each session was focused on the exploration of one theme: 1) ***turning points in the personal narrative*** – significant life events and related themes, roles, and coping resources (Chochinov et al., 2002; Keisari & Palgi, 2017; McAdams, 2001); 2) ***personal legacy*** – how the participant wished to be remembered, and what values and lessons they would like to pass on to their loved	The main activities and detailed information are provided on page 5 and also in the full protocol, which can be found in the supplementary materials**.** We also provide the name of the artists and the website where the collections of photographs can be found.	______ –

	ones (Chochinov, 2002; Testoni et al., 2021); and 3) ***future perspectives*** – desires, thoughts and concerns regarding the future and the end of life (Lang & Carstensen, 2002; Testoni et al., 2015, 2017). For the full protocol, see the **supplementary materials (Keisari et al., 2022).**<br>At the start of each session, the themes and related questions were introduced by the therapist using as prompts a set of artistic photos, selected in advance for their ability to serve as visual metaphors for that session's theme (Weiser, 2004). The full pool comprised about 80 photographs, either original images by Israeli photographers Michal Fattal and Yehudit Liberman, or licensed photos obtained from the iStock website (https://www.istockphoto.com/). Participants were asked to view the presented photographs, choose those that most reflected their own personal experiences in relation to the themes raised by the therapist, and arrange them together within a blank space on the screen. This was done using PowerPoint software, which is available on most computers and enables flexibility in the creative process of selecting, cropping, positioning, and titling digital photographs. Participants were also permitted to select and incorporate personal photographs, or other photographs taken from the Internet (all such photos were replaced by licensed photographs from the iStock website for this publication). At the end of the process, the digital photocollages were printed and sent to the participants.		
**4.**	Procedures	A full description of the procedures and activities can be found in the protocol, in the supplementary materials**.**	______________ –
	**WHO PROVIDED**		
**5.**	The sessions were conducted by the first (SK), second (SP), fourth (TE) and fifth (GM) authors, with each participant working with one therapist. The first author is a supervisor and drama therapist specializing in clinical gerontology. The second and fourth authors were doing internships in clinical psychology and drama therapy, and the fifth author was a master's degree student in clinical psychology. Two of the therapists (native Italian speakers) conducted the sessions in Italy, and two (native Hebrew speakers) in Israel.	A description of researchers/therapists is presented on page 5	______________ –
	**HOW**		
**6.**	The sessions were conducted via Zoom "share screen" function operational. and PowerPoint application.	A description of the modes of delivery and the applications we used can be found on page 5	______________ –
	**WHERE**		
**7.**	Zoom online individual video sessions in participants' homes or via computer located in the adult day care centers	A description of the settings is presented on page 5	______________ –
	**WHEN and HOW MUCH**		

**8.**	The original protocol called for three online sessions for each participant, each lasting approximately 90 minutes. Some meetings were split into two sessions to accommodate the participants. Overall, 78 sessions were conducted between December 2020 and March 2021, ranging in length from 26 to 120 minutes, with 37 sessions held in Italy and 42 in Israel. Sessions were held about 2 to 7 days apart. In addition, participants would occasionally contact the therapist outside the formal sessions, via WhatsApp or email, to share thoughts, personal photographs, texts, and ideas they wanted to incorporate into their collage. Some participants were supported by family members or day center staff in their use of technology such as setting up the computer and the Zoom meetings.	A description of the number of sessions and duration is presented on page 5	____________ _
	**TAILORING**		
**9.**	Some meetings were split into two sessions to accommodate the participants.	Personalized adaptations were made only in terms of the number of sessions, and session duration, as described on page 5	____________ _
	**MODIFICATIONS**		
**10.‡**	N/A	N/A (no modifications made)	____________ _
	**HOW WELL**		
**11.**	To ensure adherence to the protocol, the first (SK) and sixth (IT) authors conducted five training sessions with the team (2.5 hours each) prior to the intervention in order to gain familiarity and practice how to deliver the intervention. In addition, 90-minute online supervision sessions were held twice a week during the course of the study by the first author. During these sessions, the student therapists were able to discuss their work and receive professional support. Overall, 22 supervision sessions were conducted.	Strategies to maintain fidelity and adherence are described on page 5	____________ _
**12.‡**	Actual: N/A	N/A - There was no assessment of fidelity or adherence	____________ _

** **Authors** - use N/A if an item is not applicable for the intervention being described. **Reviewers** – use '?' if information about the element is not reported/not sufficiently reported.

† If the information is not provided in the primary paper, give details of where this information is available. This may include locations such as a published protocol or other published papers (provide citation details) or a website (provide the URL).

‡ If completing the TIDieR checklist for a protocol, these items are not relevant to the protocol and cannot be described until the study is complete.

* We strongly recommend using this checklist in conjunction with the TIDieR guide (see *BMJ* 2014;348:g1687) which contains an explanation and elaboration for each item.

* The focus of TIDieR is on reporting details of the intervention elements (and where relevant, comparison elements) of a study. Other elements and methodological features of studies are covered by other reporting statements and checklists and have not been duplicated as part of the TIDieR checklist. When a **randomised trial** is being reported, the TIDieR checklist should be used in conjunction with the CONSORT statement (see www.consort-statement.org) as an extension of **Item 5 of the CONSORT 2010 Statement.** When a **clinical trial protocol** is being reported, the TIDieR checklist should be used in conjunction with the SPIRIT statement as an extension of **Item 11 of the SPIRIT 2013 Statement** (see www.spirit-statement.org). For alternate study designs, TIDieR can be used in conjunction with the appropriate checklist for that study design (see www.equator-network.org).
