# Supplementary material for: Spirituality During the COVID-19 Pandemic: An Online Creative Arts Intervention With Photocollages for Older Adults in Italy and Israel
Source: Front Psychol. 2022 Jun 16;13:897158. doi: 10.3389/fpsyg.2022.897158 (PMC9245519; doi:10.3389/fpsyg.2022.897158)
Supplement: Supplementary file 2 [file Data_Sheet_2.PDF]

Supplementary material

Description of the main stages of the intervention

Stage	Main goals	Methods and tools
Stage 1: Introductory call to participants to introduce the research and its aims	• Introductory session between the therapist and the<br>• participant.<br>• Explanation of the process and the technical requirements to conduct the online sessions.	• A verbal telephone session.
Stage 2: Turning Points (Session 1)	• Creating a photocollage that visually represents turning points in the life story. Those turning points, as the most significant life-events, capture the most important themes and roles in one's narrative (Keisari & Palgi, 2017).<br>• Exploring one's most significant life-events as an opportunity to acknowledge the coping resources one has acquired in life, which can also be relevant today, while coping with the COVID-19 pandemic.	• Presenting a collection of 60 photographs. The photographs in this study were taken by the photographers Michal Fattal and Yehudit Liberman from Israel. Some of the photographs were taken from the University of Haifa's collection of photographs taken from https://www.istockphoto.com/.<br>• Participants were first asked to look at the photographs and see which photographs stimulated personal content, such as life experiences, thoughts, and feelings.<br>• Participants were then asked to look at the photographs a second time and choose between 6 to 8 that represented turning points in life or other significant memories.<br>• The therapist presented a blank space (a PowerPoint spare slide) and asked the participant how he/she would like to place the photographs within it.<br>• Participants selected one photograph at a time. They placed the photograph in the blank space and in relation to the other photographs of the photocollage. During this process they explained what the photograph represented for them to the therapist and told the stories the photographs elicited. The participants were asked to title each photograph.<br>• Once the whole photocollage had been made, the participants were asked to look at the entire photocollage. They were given time for reflection about the things they learned from these turning points in life, and the coping resources they regained. The therapist suggested that they reflect upon the relationship between these coping resources and the ways they could be useful today, while coping with the pandemic.<br>• Participants were asked about their experiences during the process of creating their photocollage and their thoughts and feeling about the product.
Stage 3: Values and Legacy (Session 2)	• Creating a photocollage that visually represents the most significant personal values in life that constitute the participants' legacy for future generations.	• Summary of the previous meeting<br>• Brief introduction to the task of the second meeting<br>• Presenting a new collection of 60 photographs that was created specifically for this session.<br>• Participants were first asked to look at the photographs to see which photographs stimulated personal content, such as life experiences, thoughts, and feelings.<br>• Participants were then asked to look at the photographs a second time and choose between 6 to 8 photographs that represented their values, as a legacy they would like to pass down to the younger generations.

		• The therapist presented a blank space (a PowerPoint spare slide) and asked the participants how they would like to position the photographs within the blank space.<br>• Participants selected one photograph at a time. They positioned the photograph in the blank space and in relation to the other photographs in the photocollage. During that process they shared what values the photograph represented for them with the therapist and told the stories that were stimulated by the photographs. The participants were asked to title each photograph.<br>• Once the whole photocollage had been made, the participants were asked to observe the entire photocollage. They were given time for reflection upon the values that were represented in the photocollage.<br>• Participants were presented the 10 values defined in Schwartz's theory of basic values (Schwartz, 2012) and some examples of these values. While looking at the photocollage they were asked to identify which Schwartz's values were present in their photocollage.<br>• The therapist suggested reflecting on the relationship between these values and the ways they can be useful today, while coping with the pandemic.<br>• Participants were asked about their experiences during the process of creating their photocollage and their thoughts and feeling regarding the product.
Stage 4: Future Perspectives and Wisdom (Session 3)	• Creating a photocollage that visually represents participants' future perspectives.<br>• Asking participants to reflect upon wisdom and how it can be helpful while coping with the COVID-19 pandemic.	• Summary of the previous meeting.<br>• Brief introduction to the task of the third meeting.<br>• Presenting a new collection of 60 photographs that was created specifically for this session.<br>• Participants were first asked to observe the photographs to see which photographs stimulated personal content, such as life experiences, thoughts and feelings.<br>• Participants were then asked to look at the photographs a second time and choose between 6 to 8 photographs that represented the way they see and wish for their and their loved ones' futures.<br>• The therapist presented a blank space (a PowerPoint spare slide) and asked the participant how he/she would like to position the photographs in the blank space.<br>• Participants selected one photograph at a time. They positioned the photograph on the blank space and in relation to the other photographs in the photocollage. During that process they shared what the photograph represented for them and their thoughts and feelings about the future that were stimulated by the photographs. The participants were asked to title each photograph.<br>• Once the whole photocollage had been made, the participants were asked to look at the entire photocollage and reflect upon their wishes for their future.<br>• Participants shared their thoughts about their own future, including end-of-life issues, and wishes for their loved ones.<br>• Participants were asked to reflect about the concept of wisdom and the way it was represented in the photocollage.<br>• The therapist suggested reflecting on the relationship between the representations of wisdom and the ways they can be useful today, while coping with the pandemic.<br>• Participants were asked about their experience during the process of creating their photocollage and their thoughts and feeling about the product.

		• Participants were asked to observe the three photocollages they created and to give a title to the whole process.<br>• Farewell.
Stage 5: Sending a hard copy of the photocollages	• Sharing the three photocollages, as a creative generative document for the participants	• Printing the three photocollages made by each participant<br>• Writing a card thanking them for participating in the study.<br>• Sending the three photocollages in an envelope to each participant.
